# Looking for variable molecular markers in the chestnut gall wasp *Dryocosmus kuriphilus*: first comparison across genes

**DOI:** 10.1038/s41598-018-23754-z

**Published:** 2018-04-04

**Authors:** Raúl Bonal, Enrique Vargas-Osuna, Juan Diego Mena, José Miguel Aparicio, María Santoro, Angela Martín

**Affiliations:** 1 Forest Research Group, INDEHESA, Escuela de Ingeniería Forestal y del Medio Natural, University of Extremadura, Plasencia, Spain; 2 DITEG Research Group, University of Castilla-La Mancha, Toledo, Spain; 3 CREAF, Cerdanyola del Vallès, Catalonia, Spain; 4 Departamento de Ciencias y Recursos Agrícolas y Forestales, Campus de Excelencia Internacional Agroalimentario (ceiA3), Universidad de Córdoba, Cordoba, Spain; 5 Grupo de Investigación de la Biodiversidad Genética y Cultural, Instituto de Investigación en Recursos Cinegéticos CSIC-UCLM-JCCM, Ciudad Real, Spain

## Abstract

The quick spread of the chestnut gall wasp *Dryocosmus kuriphilus* in Europe constitutes an outstanding example of recent human-aided biological invasion with dramatic economic losses. We screened for the first time a set of five nuclear and mitochondrial genes from *D. kuriphilus* collected in the Iberian Peninsula, and compared the sequences with those available from the native and invasive range of the species. We found no genetic variability in Iberia in none of the five genes, moreover, the three genes compared with other European samples showed no variability either. We recorded four cytochrome b haplotypes in Europe; one was genuine mitochondrial DNA and the rest nuclear copies of mitDNA (*numts*), what stresses the need of careful *in silico* analyses. The *numts* formed a separate cluster in the gene tree and at least two of them might be orthologous, what suggests that the invasion might have started with more than one individual. Our results point at a low initial population size in Europe followed by a quick population growth. Future studies assessing the expansion of this pest should include a large number of sampling sites and use powerful nuclear markers (e. g. Single Nucleotide Polymorphisms) to detect genetic variability.

## Introduction

Global trade is increasing alien species introduction all over the world, many of which are agricultural pests favoured by a poor control of the movement of plant material^1^. The accidental introduction and spread of the oriental chestnut gall wasp *Dryocosmus kuriphilus* Yasumatsu (Hymenoptera: Cynipidae) in Europe constitutes one of the most spectacular invasions detected in recent times and has already provoked dramatic economic losses in *Castanea sativa* nut production^2^.

*Dryocosmus kuriphilus* was first detected in Italian chestnuts orchards in 2002, where infested plant material brought from China was introduced^3,4^. The pest has since then literally taken Europe by storm and can be currently found in more than a dozen European countries^5^. Females oviposit into the buds and larval development provokes the abnormal development of twigs and the formation of galls^6^. The galls hamper plant growth, alter floral development and provoke reductions in chestnut yields of up to 80%^2,7^.

The spread of *D. kuriphilus* out of Italy first reached nearby countries like France and Switzerland (2005 and 2009, respectively) and then Slovenia, Croatia or Austria; nowadays, the pest has already arrived at areas as far north as the Netherlands^5^. On the eastern margin, recent records confirm its presence in Turkey^8^ and in the west it was detected in 2012 in the northeastern Iberian Peninsula^9^. In Iberia, this gall wasp is now present in most chestnut forests and orchards, including the southernmost populations of this tree in Andalusia (Spain)^10^. The rapid spread of the pest has been favoured by the movement of infested material, as the eggs are difficult to detect within the dormant buds. Also, new populations may be founded by a single female and grow in number very quickly in this fecund thelytokous species (produces fertile eggs by parthenogenesis)^11,12^.

The extremely rapid expansion of the pest over Europe suggests multiple introductions by humans^5^, as it largely exceeds the dispersal rates estimated for this insect in 8 km/year^13^. Current efforts for the control of this pest rely on the introduction of a parasitoid native from the original distribution range of the pest in China, namely *Torymus sinensis* (Hymenoptera: Torymidae), which can reduce gall wasp numbers^14^. Nonetheless, in those areas where the pest does not occur, the most urgent measure consists in avoiding its arrival. In this sense, the use of molecular techniques allows detecting *D. kuriphilu*s in infested plant material. DNA extraction from bud tissue before budburst, followed by a successful amplification of wasp genes, constitutes an undoubtable proof of the presence of wasp eggs^15^. Besides, genetic analyses may also inform about the origin of the invasive wasp populations, providing crucial information about pest dispersal aided by humans^16^.

The first molecular approach to assess the origin and spread of *D. kuriphilu*s in Europe consisted in sequencing one mitochondrial gene (cytochrome oxidase I) from wasps in Slovenia, Croatia and western Italy^5^. The presence of a unique haplotype coincident with that recorded elsewhere in Italy^17^ and identical to the most widespread haplotype in China^18^, led them to propose that the invasion resulted from a single introduction from Asia and further expansion after a severe population bottleneck^5^. This article provides valuable information on the origin of the invasion and puts forward the invasive capability of this pest. However, when mutation rates differ across genes, as is the case in the fast-evolving hymenoptera mitochondrial genome^19,20^, sequencing more than one gene could show intra-specific genetic variability otherwise undetected.

In the present study, we sequenced and screened five genes (mitochondrial and nuclear) in an introduced population of the oriental chestnut gall wasp in Andalusia (southern Spain), far away from the origin of the invasion in Europe. Our specific objectives were: i) to analyse the intra-specific genetic variability of each gene among the Spanish samples ii) to compare the Iberian sequences with those available from Europe and Asia iii) to assess which of these genes could be suitable (i. e. variable enough) markers for future studies on the phylogeography and population genetics of the species.

## Results

Only one haplotype was retrieved for each of the nuclear genes sequenced (28S and ITS2). The 28S (D3-D5 region) Iberian haplotype (Accession number MH116002) was identical to that obtained from a *D. kuriphilus* individual collected in Italy (Accession number DQ286819). In the case of ITS2, the Iberian haplotype (Accession number MH116003) showed no differences with those reported from Japan and Italy (Accession numbers AB200276 and JQ229194, respectively). No double peaks were detected at any site after a careful inspection of the chromatograms.

**Figure 1:**
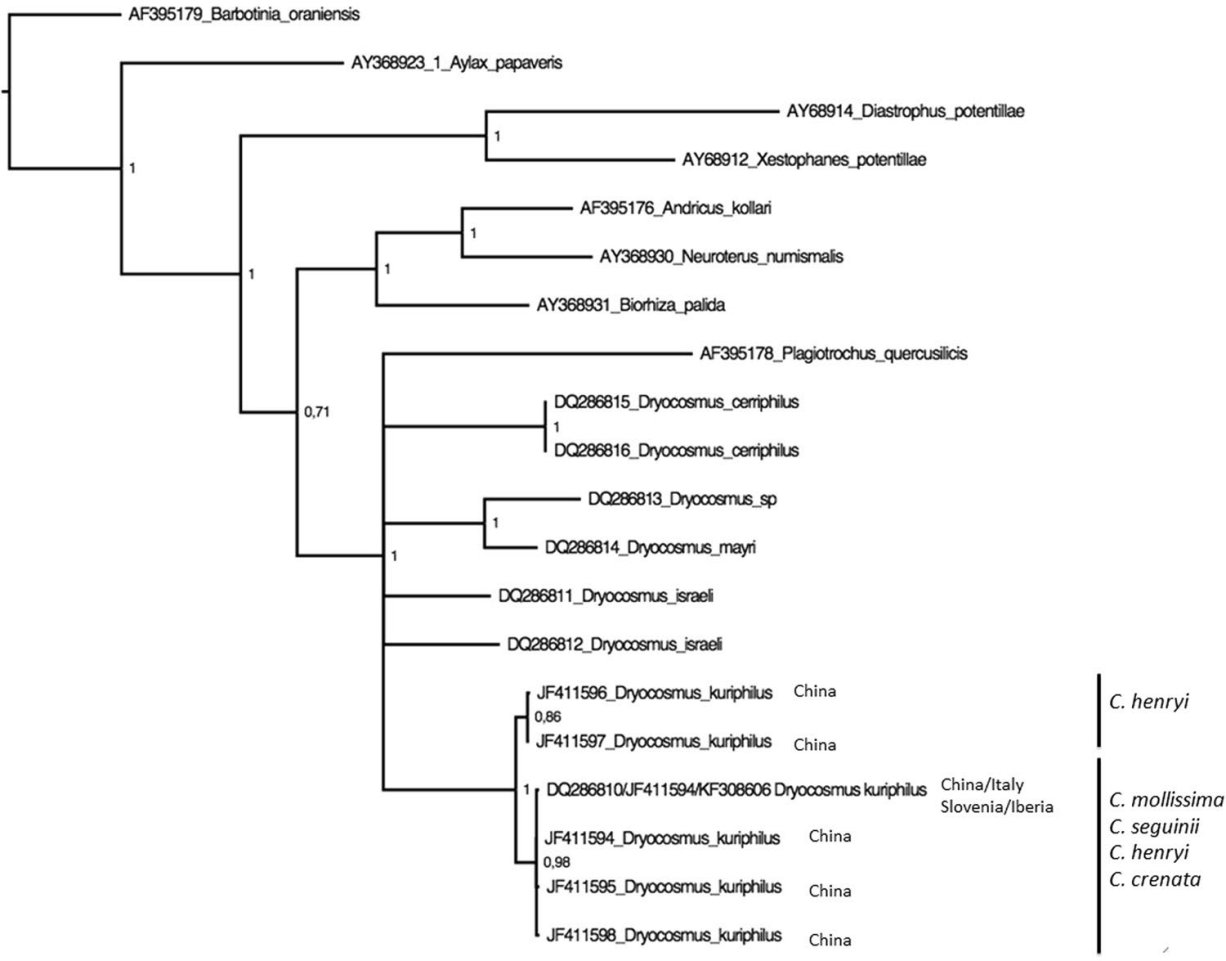
Cytocrome oxidase I gene tree for *Dryocosmus spp*. and allied genera (sequences available from Ács *et al*. (2007, ref.^17^). Tree topology was inferred using Bayesian inference with substitution models HKY+ Gamma for the first and third codon positions and F81+ inv for the second one. Support for each node is represented by the Bayesian probability value. Taxonomic identity of each sequence taken from GenBank, numbers besides scientific names indicate the accession numbers.

The sequence of cytochrome oxidase I (hereon cox1) was identical in the 24 individuals analysed (Accession number MH119939) and, at the same time, showed no differences with those previously reported from Italy and Slovenia (Accession numbers DQ286810 and KF308606) and with one of the haplotypes in China (JF411594). The cox1 gene tree built for *Dryocosmus spp*. and allied genera showed that *D. kuriphilus* constitutes a monophyletic clade (Fig. 1). Within this, *D. kuriphilus* sequences formed two clusters, one of them with wasps collected only within galls of *Castanea henryi*^21^. The Iberian haplotype was included in the other clade, which grouped sequences from individuals recorded on different species of *Castanea* (Fig. 1). All the sequences of the mitochondrial 16S (Accession number MH116001) were identical.

The case of cytochrome b (hereon cytb) deserves special attention: from the 24 Iberian wasp larvae we obtained two distinct haplotypes; the first one (hereon Iberia 1) was the most prevalent (Accession number MH119938), 18 individuals versus 6 that beared the haplotype Iberia 2 (Accession number MH119937). The two haplotypes were found in the three nearby sampling sites, and their relative prevalence did not differ among sites (Chisq = 0.33; df = 2; P = 0.84). According to the uncorrected genetic distance, the divergence between these two haplotypes was 12% (34 variable sites in a sequence 275 bp long) (Table 1a) and 13.2% applying the Kimura 2-parameter model (Table 1b). We repeated the PCRs and the sequencing to discard any potential error at any stage of the process. We confirmed that no error was made, the sequences were identical to the original and the same two distinct haplotypes were retrieved.

**Table 1:** Pairwise genetic distances between the cytochrome b sequences recorded in Iberia, Italy (ITAL) and Hungary (HUN).

	DQ286803_ITA	IBERIA 2	KU760838_ITA	KU760839_HUN	IBERIA 1
**a**					
DQ286803_ITA	0				
IBERIA 2	1	0			
KU760838_ITA	20	21	0		
KU760839_HUN	20	21	0	0	
IBERIA 1	34	33	34	34	0
**b**					
DQ286803_ITA	0				
IBERIA 2	0.003				
KU760838_ITA	0.077	0.081			
KU760839_HUN	0.077	0.081	0		
IBERIA 1	0.136	0.132	0.136	0.136	0

There were a total of 275 positions in the final alignment dataset. The upper panel (Table 1a) shows the uncorrected genetic distance (number of variable sites) and the lower one (Table 1b) the distance calculated using the Kimura 2-parameter model^45^.

**Figure 2:**
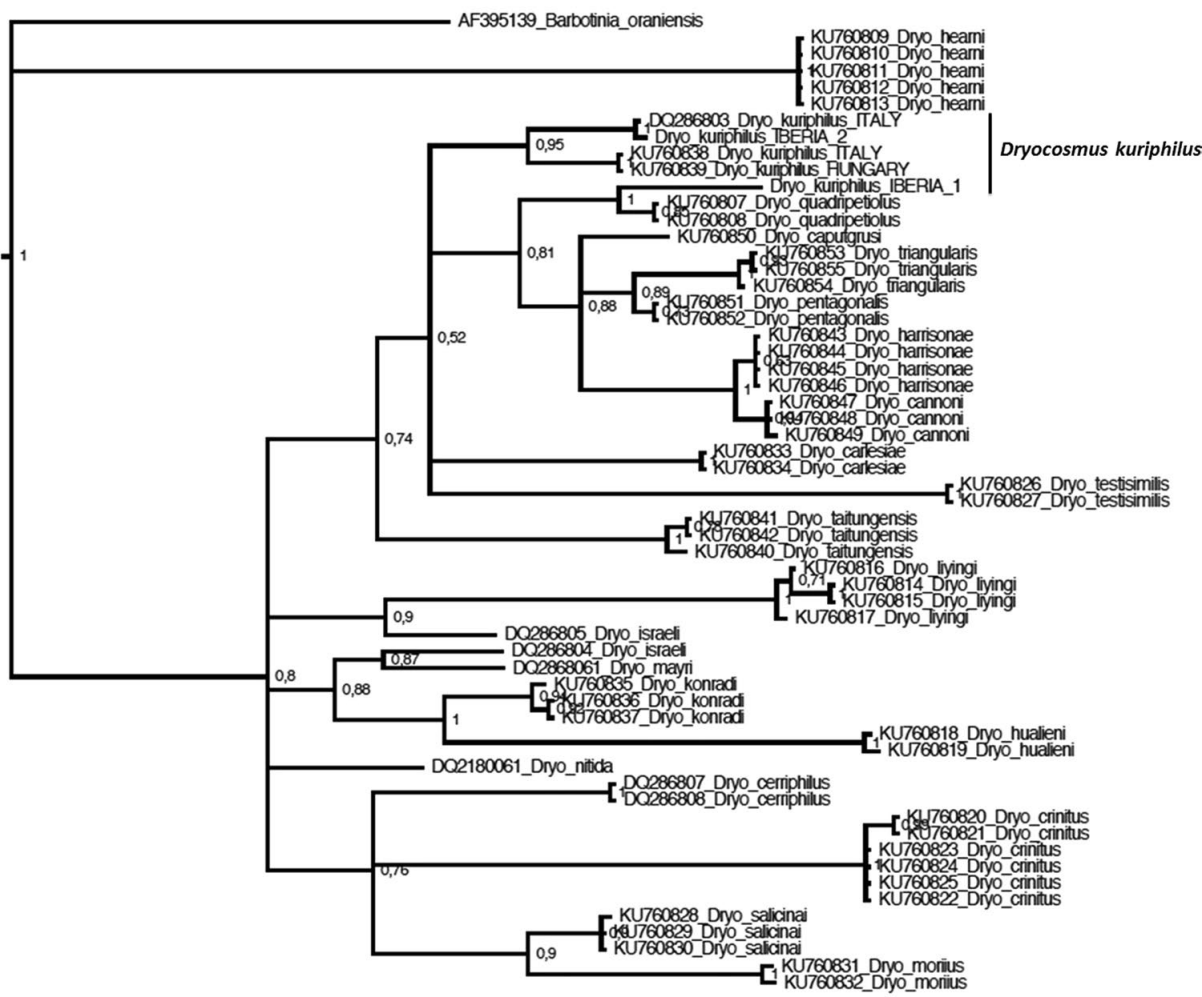
Cytocrome b gene tree including all the sequences available for *Dryocosmus* spp. Tree topology was inferred using Bayesian inference with the substitution model HKY+ Gamma for the three codon positions. Support for each node is represented by the Bayesian probability value. Taxonomic identity of each sequence taken from GenBank, numbers besides scientific names indicate the accession numbers.

When we built the gene tree with all the cytb sequences available for *Dryocosmus spp*. (Fig. 2), we realised that such an extreme intra-specific divergence was not uncommon. From GenBank we downloaded three more sequences that corresponded to two highly divergent haplotypes: the first one was recorded in Italy (reference DQ286803) and the second in Italy and Hungary (references KU760838 and KU760839, respectively). They diverged more than 7% using either the uncorrected genetic distance or the K2P model (Table 1a and b). One of these haplotypes (DQ286803) clustered with Iberia 2 (from which it diverged in only one base located in the third codon position) (Table 1a and b; Fig. 2). Within the *Dryocosmus* spp. cytb gene tree these three haplotypes (four sequences) formed a monophyletic clade with a high node support (Fig. 2). By contrast, the haplotype Iberia 1 of *D. kuriphilus* grouped with *Dryocosmus quadripetiolus* (Fig. 2) and the K2P genetic divergence between them was 6.1%.

Due to the striking genetic divergence among cytb haplotypes we conducted a series of tests to detect potential nuclear mitochondrial pseudogenes (*numts*). First, we inspected carefully the 24 cytb chromatograms of the Iberian samples and in none of them there were double peaks. Moreover, when the cytb haplotypes (the two Iberian and the other two recorded in Italy and Hungary) were translated into aminoacids in none of them there were any stop codons; no indels were found either. Yet, this is not enough to discard the existence of *numts*^22^. The GC content of the *D. kuriphilus* haplotypes ranged from 22.5 to 22.9%, what falls within the limits expected for mitochondrial DNA^22^. However, the mutation rates and patterns of the *D. kuriphilus* haplotypes grouped in the independent cluster (all except Iberia 1) (Fig. 2) deviated significantly from the reference values, thereby showing that they could be nuclear copies of mitochondrial genes. The pairwise comparison between the haplotype collected in Italy and Hungary and the haplotype Iberia 2 (we did not include the Italian DQ286803 because the sequence was almost identical to Iberia 2) showed that mutation rates in the second codon position exceeded by far the values expected in mitochondrial DNA (Table 2). For this reason, the relative frequency of nonsynonymous substitutions also exhibited extremely high values. Moreover, the relative frequencies of transversions and transitions in the third codon position did not agree with those reported for mitochondrial DNA, they were too high and too low respectively (Table 2). We got similar results when we compared the *D. kuriphilus* haplotypes of the separate cluster with the haplotype Iberia 1 or the closely related species *Dryocosmus quadripetiolus* (Table 2; Fig. 2). By contrast, when the sequences of the haplotype Iberia 1 and *Dryocosmus quadripetiolus* were compared, mutation rates agreed with those expected for mitochondrial DNA (Table 2) in all the parametres, with just a very slight deviation in the case of transversions and nonsynonimous substitution rates.

**Table 2:** *In silico* analyses of three *Dryocosmus kuriphilus* cytochrome b haplotypes (pseudogenes: Haplotype Iberia 2 and KU760839) and genuine mitochondrial DNA (Haplotype Iberia 1).

	Haplotype Iberia_1 vs D.quadripetiolus	Haplotype Iberia_2 vs KU760839	Haplotype Iberia_2 vs D.quadripetiolus	KU760839 vs D.quadripetiolus	Expected value for mtDNA
1st codon pos. substitutions	11	22	17	15	14.9 ± 9.4%
2nd codon pos. substitutions	6	16	20	25	4.5 ± 3.5%
3rd codon pos. substitutions	83	62	63	60	80.6 ± 21
Transitions (3rd codon position)	73	36	57	35	84.9 ± 18.1
Transversion (3rd codon position)	27	64	43	65	15.1 ± 7.6%
Nonsynonimous substitutions	16	33	36	33	7.47 ± 5.4%

Mutation rates (percentage for each codon position) and the relative frequency of transitions, transversions and nonsynonimous substitutions are shown for pairwise comparisons between the two pseudogenes and between each *D. kuriphilus* sequence and a reference mitDNA from the closely related *D. quadripetiolus*. The last column shows the expected mitDNA values with a confidence interval at α = 0.05^22^.

## Discussion

Our comparison across-genes evidences the extremely low genetic diversity of the populations of the invasive *D. kuriphilus* in Europe, according with previous studies based on a single-gene^5^. The null intra-specific divergence of the nuclear 28S rDNA agrees with the slow mutation rates of this gene, which has shown little or no variability even between hymenoptera species of the same genus^17^. The gene ITS2 showed no variability either, despite nuclear internal transcribed spacers have shown inter-population variability in bees^23^. Moreover, we found no indels or nucleotide substitutions (absence of double peaks in the chromatograms) like those found in other insect species^24^. ITS2 homozigosity in *D. kuriphilus* is not surprising considering that one route to ITS intra-individual variability is the combination of different haplotypes inherited via sexual reproduction^25^ and this wasp is thelytokous (fertile eggs by parthenogenesis). However, variability might still arise through mutations, as in other species multiple clones of this gene may be found in the same individual^24^. The lack of variability could thus support the hypothesis on the low number of founders in the invasive populations.

The unique haplotype of the mitochondrial gene cox1 retrieved in the Iberian samples was shared with all the chestnut gall wasps sequenced from central Europe and northern Italy^5^ and with one of the six haplotypes recorded in China^18^. The gene tree including all the sequences labelled as *D. kuriphilus* in GenBank depicted two clusters with little genetic variability within each. The haplotype found in Europe for *C. sativa* grouped with haplotypes retrieved from wasps feeding on different species of *Castanea* spp.: *C. mollissima*, *C. henryi*, and *C. seguinii* in China^18^. The second cluster corresponded to wasps collected only on *C. henryi*^21^. The existence of cox1 haplotypes with a higher genetic divergence with respect to the rest had been reported before^5,18^, but their phylogenetic relatedness remained unknown. The present gene tree shows that they form a monophyletic group that would correspond to *Dryocosmus zhuili*, a new species that feeds only on *C. henryi* recently described on a morphological basis^21^. The absence of *C. henryi* in Europe would hinder the invasion by this more specialised species. By contrast, the wider trophic range of *D. kuriphilus* in its native range (it has been recorded on different host trees: *C. crenata*, *C. mollissima*, *C. henryi*, and *C. seguinii*)^26^ could have facilitated the colonization of a new host tree (*C. sativa*) not present in China, as a wide trophic niche generally favours pests^27^.

The European haplotype of cox1 was the most widespread one in China^18^. The arrival of the most frequent haplotype in the native distribution range to the invaded areas reflects a typical founder effect^28^. The rare alleles are less likely to reach the new populations founded after an invasion event, resulting in a decrease of the overall genetic diversity^29^. The mitochondrial gene 16S also showed null genetic variability but, as we sequenced it for the first time in the genus *Dryocosmus*, we could not compare it with any sequence from elsewhere in the native or invasive distribution range.

The striking intra-specific genetic divergence in cytb was provoked by the presence of pseudogenes. The genetic distance between the two *D. kuriphilus* cytb haplotypes recorded in Iberia largely exceeds the usual intra-specific genetic divergence reported for other *Dryocosmus* spp.^26^. Such intraspecific divergence also exists in the invasive populations of Italy and Hungary, but it had gone unnoticed because the sequences were reported by two different studies^17,26^. These values are also extremely high compared to the intraspecific variability reported for other mitochondrial genes (e. g. cytochrome oxidase I), which was generally below 1%^30,31^.

Exaggerated intra-specific genetic divergence in a mitochondrial gene often indicates the existence of mitochondrial nuclear insertions (*numts*)^32^; however, the sequences did not fulfil some of their characteristics. The typical double peaks in the chromatograms and their combination forming chimeric sequences^33,34^ were not detected, moreover, no stop codons or indels were found in any of the four haplotypes either. Nonetheless, further analyses showed that some of those haplotypes were really pseudogenes. Three of them formed a separate clade in the cytochrome b gene tree, whereas the remaining one (haplotype Iberia 1) was closer to other species of *Dryocosmus* (Fig. 2). The elevated rates in the second codon position, the proportions of transitions/transversions in the third codon position and the high rate of nonsynonymous substitutions showed that the haplotypes in the separate clusters were nuclear copies of the mitochondrial gene (numts). Only the haplotype Iberia 1 was genuine mitochondrial DNA.

The pseudogenes found in *D. kuriphilus* share some characteristics with those reported in other organisms. Studies with small mammals have shown that cytb pseudogenes also form separate clades in the gene trees^35^. Likewise, *numts* usually appear in the phylogeny in a basal position, with shorter branches from the common ancestor compared to the mitochondrial gene and closer to sister species^33–35^.

The infection by *Wolbachia*, may account for the high genetic divergence recorded in some species of insects^36^, yet it cannot explain our results. *Wolbachia* is a symbiont intracellular bacteria maternally inherited that increases the fitness of the females that bear it^37^. It has also male-killing abilities and promotes parthenogenetic reproduction, what favours the rapid spread of the haplotypes infected by *Wolbachia* thereby reducing the genetic diversity of the populations. Extreme intra-specific genetic divergence may occur when there is a *Wolbachia*-driven introgression. If a male mates with a female of a closely related species infected by *Wolbachia* and the offspring females are fertile, a new and very divergent mitochondrial haplotype could spread if the hybrid female backcrosses with the father’s species^36^. In *Dryocosmus kuriphilus Wolbachia* infection has been reported in some populations, but no males have been recorded so far^11^, what excludes this introgression scenario. Furthermore, as mitochondrial DNA does not recombine, such divergence should exist in all mitochondrial genes^38^ and it only occurs in cytb.

The detection of pseudogenes in *D. kuriphilus* recommends caution before drawing any conclusion in phylogenetic and population genetic studies. In fact, the sequences reported by other studies and uploaded to GenBank turned out to be pseudogenes^17,25^. In our study site we recorded both genuine mitochondrial DNA and pseudogenes, if well the real mitochondrial cytb was sequenced in the majority of the individuals (75%). We stress the need of performing *in silico* analyses of the sequences^22^ to detect them and interpret the results properly.

Despite their potential confounding effects, the mitochondrial copies of mitochondrial genes (*numts*) may provide interesting information about the species demography^34^. Previous studies have shown that clusters of *numts* may be orthologous^34^, that is, they would result from an ancient insertion of mtDNA in the nuclear genome and subsequent duplications. This might be the case of at least two of the pseudogene haplotypes recorded in our study (Iberia 1 and the Italian DQ286803), as they differ in only one base. If they were orthologous, we could not state that there have been multiple introductions in Europe from its native range, but at least say that not all the *D. kuriphilus* are clones because the invasion might have started with more than one individual.

## Conclusion

*Dryocosmus kuriphilus* exhibits a very low genetic diversity, probably favoured by its strict parthenogenetic reproduction and the infection of *Wolbachia* (in the case of mtDNA)^11^. In its European invasive range genetic diversity is even lower as a consequence of the founder effect. Nuclear markers with low mutation rates (28S and ITS) and mitochondrial genes (citox1, cytb and 16S) have no variability in Europe. In the case of cytb we detected one pseudogene in some individuals from our study sites and two more from other European localities. The existence of pseudogenes stresses the need of performing careful *in silico* sequence analyses when working with mitDNA in this species. The pseudogenes may provide interesting information, though. Two of them might be orthologous and, if so, the invasion might have started with more than one individual, not all chestnut gall wasps in Europe would be clones. Future studies including more localities within the European invasive range and using preferably highly variable nuclear markers such as SNPs (Single Nucleotide Polymorphisms) will show the expansion routes of this pest that is provoking dramatic economic losses.

## Methods

### Study area and species

The study was carried out in the Refugio del Juanar, locality of Marbella (province of Málaga, 36° 34.79′ N 04° 53.13′ O, 820 m.s.l). In this area it is possible to find naturalized chestnut stands forming relict woodlands, as well as orchards for nut production, being an important economic resource for local farmers^39^.

The chestnut gall wasp *D. kuriphilus* (Hymenoptera: Cynipidae) is a species native to Asia that was accidentally introduced in Italy in 2002^3^. In the Iberian Peninsula it was first detected in the northeast in 2012^9^; in the study area the first record dates from 2014. *D. kuriphilus* is an univoltine species, females oviposit into the buds in early summer dying afterwards. Larvae hatch and overwinter within the dormant buds until the following spring. Budburst starts in April in our study area, and the new branchlets that develop from infested buds grow abnormally forming fleshy galls on the shoots and leaves. Larvae feed and grow within those galls for approximately a month and then pupate, more than one larva are usually found together within the same gall. The fully developed adults drill an exit emergence hole to leave the gall in June^15^, then they fly and look for new buds to oviposit. Females do not need to copulate as they are telythokous; parthenogenesis is the only reproductive strategy known and to date as only females have been found^11^.

### Sampling

Samples were collected during April and May 2015 in the area where the pest was first detected in southern Spain. Grown galls were picked from the trees in three nearby sites distant approximately 100 m (36° 34.7′ N – 36° 34.9′ N; 4° 53.1′ O – 4° 53.3′ O). In total, 389 galls were taken to the laboratory and opened to extract the larvae, which were immediately kept in ethanol 100% for further molecular analyses. From these larvae we randomly selected 24 (8 from each sampling site), which were taken to the genetics laboratory for DNA extraction and sequencing. The comparison of larval DNA with published reference sequences from previously determined individuals has proved as a very useful method of identification and assessment of unequivocal trophic relationships between plants and insects^40^.

### Laboratory methods

DNA was extracted from larval tissue according to the salt extraction protocol^41^. For each individual we then amplified a total of five genes, two nuclear (28S and ITS2) and three mitochondrial (cytochrome oxidase I, cytochrome b and 16S). We chose those genes because for most of them (with the exception of 16S) there were reference sequences in GenBank to compare with. Also, mitochondrial DNA mutation rates are higher than those of nuclear DNA and intraspecific genetic divergence could differ between these two types of markers^15^.

For the nuclear gene 28S rDNA (D3-D5 region) and the mitochondrial DNA coding for the protein cytochrome b we used the primers and PCR protocols described in Sartor *et al*. (2012, ref.^15^); for the nuclear ITS2 (ribosomal Internal Transcribed Spacer region 2), located between the genes coding for the 5.8S and 28S rRNA, we followed Ji *et al*. (2003, ref.^42^). In the case of the mitochondrial 16S, coding for the large ribosomal subunit (16S rRNA), we employed general primers that have been used in a wide range of arthropods^43^. By last, the fragment of the mitochondrial gene cytochrome oxidase I, commonly used as universal DNA barcode, was amplified using the primers LEP (R1) and LEP (F1)^44^.

Sequencing was run on a 3730XL DNA analyser and sequences were edited using SEQUENCHER 4.1 (Gene Codes Corp., Ann Arbor, MI, USA). The chromatograms were carefully inspected to detect double peaks that, in the case of mitochondrial DNA, could be indicating the presence of nuclear insertions of mitochondrial DNA segments (so called *numts*)^31,32^ and, in nuclear genes, heterozygosity at a certain locus or multiple copies of the same gene^23,24^. All sequences were stored at the public repository GenBank.

When the edition was over the sequences of all genes, with the exception of the mitochondrial 16S rDNA, were aligned with all the haplotypes available at GenBank for each one. Alignments were created using CLUSTALW supplied via http://align.genome.jp (gap open and gap extension penalties were those provided by default by the software, 15 and 6.66 respectively). Sequences were trimmed on the extremes to reduce the proportion of missing data (see alignment lengths in Table 3). The alignment matrix of each gene was inspected using MacClade software version 4 to detect variable sites^45^. Both cox1 and cytb were translated into to aminoacids with a double objective: i) test for the presence of stop codons in the middle of the sequences of these intronless functional genes, as that would be also an indication of the presence of numts^31,32^, and ii) calculate the codon positions for further bayesian phylogenetic inference.

**Table 3:** Length of the sequence alignments (number of base pairs) of each of the genes screened in this study.

Gene	Alignment length
**Mitochondrial Genes**	
Cytochrome Oxidase I	641
Cytochrome b	275
16S ribosomal DNA	366
**Nuclear Genes**	
28S ribosomal DNA	270
Internal Transcribed Spacer Region 2	455

For the genes showing any variability we calculated the pairwise genetic distance using the software MEGA7^46^. The distance between sequences was computed following two methods: i) the Kimura 2 Parameters model (K2P)^47^ and ii) the uncorrected genetic distance (total number of variable sites over the whole DNA sequence). We also used MEGA 7 to assess mutation rates, transitions/tranversions and nonsynonymous substitution rates in the *in silico* analyses to detect potential nuclear copies of mitochondrial DNA^22^.

We built two separate gene trees based on cytochrome oxidase I and cytochrome b. We did so to assess the phylogenetic position of the Iberian haplotypes of *D. kuriphilus* with respect to haplotypes of the same species from other geographic areas and also with respect to other species of the same or allied genera. We chose those genes due to the higher number of sequences available at GenBank. For cytochrome oxidase I we pooled the Iberian *D. kuriphilus* sequences with those reported from China^18^, Central Europe^5^ and Italy^17^. This last study also provided sequences from other allied genera of gall wasps that were included in the gene tree. In the case of cytochrome b we built the tree using exclusively sequences of species within the genus *Dryocosmus*. This was possible due to the availability of cytochrome b sequences for a total of 24 *Dryocosmus* spp.^17,25^.

Gene trees were built following Bayesian inference using Mr Bayes 3.2^48^ applying the substitution model estimated by Partition Finder 1.1.1^49^. The best substitution models were assessed by Bayesian Information Criterion (BIC): for cytochrome oxidase I one partition was defined for each of the three codon positions, being the substitution model HKY + Gamma for the first and third and F81 + inv for the second position. In the case of cytochrome b, it grouped the 1st and 2nd codon positions on one side and left the 3^rd^ in a separate partition; HKY + Gamma was the model selected in both of them. For the Bayesian inference two parallel runs of 10 million generations each were conducted using one cold and two incrementally heated Markov chains (Λ = 0.2), sampling every 1,000 steps. We first checked the standard convergence diagnostics implemented in MrBayes and then assessed the average standard deviation of the split frequencies to deduce that the Markov chain had reached stationarity. After 500,000 generations, the average standard deviation of the split frequencies stabilized in values close to zero (0.001). Hence, phylogenetic trees were summarized using the all-compatible consensus command with 25% burn-in. In the two gene trees *Barbotinia oranensis* was used as outgroup (as in Ács *et al*. (2007, ref.^17^).
